# Commentary: Brain-to-Brain Synchrony Tracks Real-World Dynamic Group Interactions in the Classroom and Cognitive Neuroscience: Synchronizing Brains in the Classroom

**DOI:** 10.3389/fnhum.2017.00554

**Published:** 2017-11-21

**Authors:** Francisco J. Parada, Alejandra Rossi

**Affiliations:** Laboratorio de Neurociencia Cognitiva y Social, Facultad de Psicología, Diego Portales University, Santiago, Chile

**Keywords:** EEG, hyperscanning, research technology, data acquisition, embodied cognition, social neuroscience

Earlier this year, Dikker et al. (2017) published a research report opening an important new chapter in the ongoing dialogue between Neuroscience and Education, namely, the possibility of successfully acquiring and analyzing diachronic human neurophysiological data during semi-structured, real-world classroom interactions using low-cost data-acquisition technologies. Taken as disciplinary work [i.e. hyperscanning (e.g., Astolfi et al., 2010) or neuroeducational (e.g., Mercier and Charland, 2013) studies], the article surely deserves praise; however, as noted by Bhattacharya (2017) it also signifies a milestone for the entire field of Cognitive Neuroscience as it represents a big step in settling a paradigm shift, which has been slowly gaining grounds in our discipline. Given the excitement that the concept of a “paradigm shift” can bring out along with the new research avenues that low-cost technologies offer, in this commentary we offer a perspective about the challenges and implications of the ever-increasing possibility of studying cognition in its “natural state.”

Social Neuroscience is an exciting new field that has provided in-depth insights, expanded the range of topics, and added an unprecedented layer of complexity to the understanding of the neural basis of human behavior. Thanks to this multidisciplinary effort, researchers interested in the neural basis of human behavior have explored not only how traditionally-conceptualized “lower-level” and “higher-level” cognitive processes relate to specific brain structures or concerted neural dynamics, but also have turned to explore how the brain supports other mental processes that are key in the interaction between individuals. However, the field still presents some important limitations at the theoretical and methodological levels. Most social cognition studies investigate behavior and concomitant brain activity of isolated individuals while exposed to stimuli of social relevance (e.g., facial and/or bodily gestures as in Thompson et al., 2007), social nature (e.g., facial expression of emotions as in Vuilleumier and Pourtois, 2007), or immerse in a social context (e.g., increased number of individuals within the stimuli as in Akitsuki and Decety, 2009; Puce et al., 2013) without actually involving a *real interaction* with another person (i.e., simulated interactions as in Caruana et al., 2016). These approaches are commonly referred to as “one-person-neuroscience.” Most experiments use experimental paradigms probing “offline” social cognition, where isolated participants have to think about other people's mental states while being detached from a real social interaction (Pfeiffer et al., 2013).

Without a doubt, these approaches have advanced the field tremendously, but have also limited the exploration of *real life* social cognition; when information is diachronically and mutually exchanged within an appropriate temporal frame between individuals. It is within this context that it has been suggested that in order to further develop social neuroscience as a field and gain more knowledge about the neural basis of social interaction, researchers should move toward quantifying the interpersonal co-regulated coupling between interacting partners (Goldman and de Vignemont, 2009), while they mutually and continuously affect one another (Varela et al., 1991; Clark, 2008, 2013), either by mutual coordination or cooperation (Konvalinka and Roepstorff, 2012). The work by Dikker et al. (2017) embodies the aforementioned spirit.

Thus, in order to better study the neural basis of situated and embodied social interaction, we suggest that each study contributing to the referred *paradigmatic shift* should be evaluated (*a priori* or *a posteriori*) considering two main challenges depicted in Figure 1:

- The *theoretical need* of developing a coherent framework for interpreting results and mapping the whole spectrum of relations between personal, social, and neural dynamics.
- The *methodological need*, referring to the relationship between the ever-improving data-acquisition technologies and the actual usage plausibility of such technologies at a single-subject and/or group level. The latter can be further divided into a *technical requirement* of developing novel experimental setups to observe and measure interactions between two or more people and an *analytical requirement* of generating novel analysis procedures to quantify mutual interactions.

**Figure 1:**
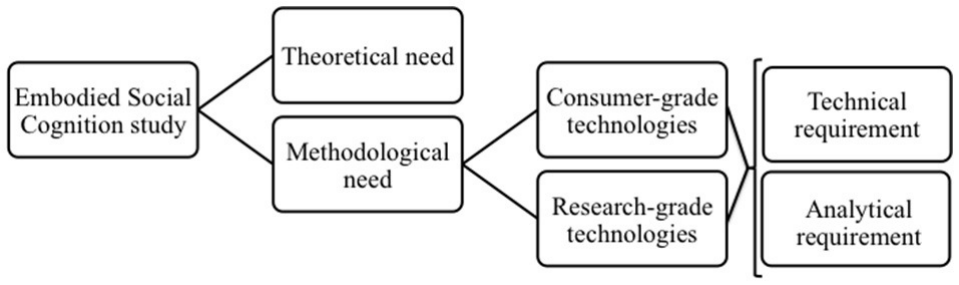
When studying the embodied nature of social cognition, regardless of how close or far away a research design is from “synthetic laboratory settings,” it must consider theoretical and methodological needs.

Although the field has been energetically revisiting and discussing about the *theoretical* need (e.g., Konvalinka and Roepstorff, 2012; De Jaegher et al., 2016; Krakauer et al., 2017), at this early stage, it is important to avoid the idea that the *methodological need* can be considered a “won battle” thanks to economically-accessible *consumer-grade technologies* (Bhattacharya, 2017). Recent evidence indicates that although the current technical landscape is very promising, we must proceed with caution (Melnik et al., 2017). Thus, we would like to point out that “more naturalistic” data acquisition can be equally undertaken using both *consumer-grade*, as in Dikker et al. (2017), as well as *research-grade technologies*, each providing advantages and disadvantages. On the one hand, *consumer-grade* equipment allows to concomitantly acquiring data from larger samples of subjects while decreasing signal quality and hindering analysis possibilities. On the other hand, *research-grade technologies* allow far more complex analyses but at a major set-up complexity and economical cost. Finally, we would like to stress that, contrary to Bhattacharya (2017), we believe *consumer-grade technologies* are not the natural next step outside “synthetic laboratory settings,” but a parallel path facing similar, if not larger, *technical* and *analytical requirements* that *research-grade technologies* confront (Figure 1). Therefore, in times of paradigmatic change, with new and exciting theoretical vistas encompassed within the *embodied social cognition* framework and new available technologies, social neuroscience wants to be methodologically cautious and remember that research questions and epistemological views guide our work. We want to *extend* the laboratory toward the real world, not *escape* from it.

## Author contributions

FP conceptualized and wrote the commentary. AR discussed the original idea and edited the commentary.

### Conflict of interest statement

The authors declare that the research was conducted in the absence of any commercial or financial relationships that could be construed as a potential conflict of interest.
